# Artificial Intelligence and Machine Learning in Bone Metastasis Management: A Narrative Review

**DOI:** 10.3390/curroncol33010065

**Published:** 2026-01-22

**Authors:** Halil Bulut, Serdar Demiröz, Enes Kanay, Korhan Ozkan, Costantino Errani

**Affiliations:** 1 Cerrahpasa School of Medicine, Istanbul University Cerrahpasa, 34098 Istanbul, Turkey; 2 Department of Orthopaedics and Traumatology, Kocaeli University, 41001 Kocaeli, Turkey; 3 Department of Orthopaedics and Traumatology, Acibadem Atasehir Hospital, 34642 Istanbul, Turkey; 4 Orthopaedics and Traumatology, Instituto Ortopedico Rizzoli, 40136 Bologna, Italy

**Keywords:** artificial intelligence, bone metastasis, machine learning, prognostic modeling, orthopedic oncology

## Abstract

Background: Artificial intelligence (AI) and machine learning (ML) are increasingly used in the diagnosis and management of bone metastases, spanning lesion detection, segmentation, prognostic modeling, fracture risk assessment, and surgical decision support. However, the literature is heterogeneous and rapidly evolving, making it difficult for clinicians to contextualize these developments. Methods: We performed a narrative review of the literature on AI/ML applications in bone metastasis management, focusing on studies that address clinically relevant problems such as detection and segmentation of metastatic lesions, prediction of skeletal-related events and survival, and support for reconstructive decision-making. We prioritized recent, peer-reviewed work that reports model performance and highlights opportunities for clinical translation. Results: Most published studies center on imaging-based diagnosis and lesion segmentation using radiomics and deep learning, with generally high internal performance but limited external validation. Emerging work explores prognostic models and biomechanically informed fracture risk estimation, yet these remain at an early proof-of-concept stage. Very few frameworks are integrated into routine workflows, and explainability, bias mitigation, and health-economic impacts are rarely evaluated. Conclusions: AI and ML tools have substantial potential to standardize imaging assessment, refine risk stratification, and ultimately support personalized management of bone metastases. Future research should focus on externally validated, multimodal models; development of AI-augmented alternatives to the Mirels score; federated multicenter collaboration; and routine incorporation of explainability and cost-effectiveness analyses.

## Simple Summary

Bone metastases are a major source of pain, pathological fracture, and loss of function in patients with advanced cancer, yet clinical decision-making still relies heavily on subjective image interpretation and empirical tools such as the Mirels scoring system. This narrative review synthesizes current work on how artificial intelligence and machine learning can support orthopedic surgeons and oncologists along the entire metastatic bone disease pathway. We describe applications that automatically detect and segment bone lesions on computed tomography, magnetic resonance imaging, and nuclear medicine studies; quantify bone quality and lesion burden; estimate fracture risk using imaging and biomechanical features; and predict survival and functional outcomes after radiotherapy or surgical reconstruction. We also highlight key limitations, including small, single-center cohorts, lack of external validation, poor integration into picture archiving and communication systems, limited interpretability, and absence of health economic evaluation. We conclude by outlining practical research priorities, such as developing transparent, prospectively validated fracture risk and prognostic models that can meaningfully improve patient selection, optimize implant choice, and reduce skeletal-related events in real-world practice.

## 1. Introduction

Bone metastases are a significant clinical and public health issue, impacting approximately one million patients worldwide annually [1,2]. Their prevalence continues to rise, largely due to improved cancer survival and aging populations. In the United States, about 5% of all cancer patients develop bone metastases [3]. Prostate and breast cancers are the most common causes, with rates as high as 30% [4]. SREs, especially pathological fractures, continue to be a significant cause of morbidity, resulting in intense pain, decreased mobility, compromised functional independence, and a lower quality of life [4,5,6,7]. These complications also put a lot of stress on patients and healthcare systems, both financially and mentally [6,7].

Preventing pathological fractures in metastatic long bones is still difficult because of the size of the lesions, their location in the body, the quality of the bones, and the conditions under which they are loaded [8,9,10,11,12,13,14]. Even though radiographic and clinical evaluations are used a lot, the current tests do not have standardized and quantitative predictive metrics [8,9,10,11,12,13,14,15]. The Mirels’ scoring system, although widely utilized, is characterized by inadequate sensitivity, specificity, and reproducibility [16]. As a result, there is a pressing necessity for more objective and personalized instruments to forecast fracture risk and facilitate prompt surgical intervention.

In metastatic bone disease, decision-making goes beyond just predicting fractures. It also includes accurate diagnosis, prognosis, and personalized surgical planning, all of which need to combine different types of clinical and imaging data. This complexity has led to increased interest in computational techniques that can combine different types of data. AI and ML have become promising tools in musculoskeletal oncology because they can process multimodal inputs—radiological, biomechanical, laboratory, and clinical—to find subtle, non-linear patterns that humans cannot see [17,18,19,20,21]. Preliminary studies suggest that algorithms, including convolutional neural networks, transformers, and ensemble learning models, may surpass traditional statistical techniques in functions such as fracture risk assessment, survival forecasting, and surgical planning [18,19]. Nonetheless, significant challenges persist concerning clinical validation, interpretability, data standardization, and practical implementation.

While numerous reviews have examined AI in general oncology or soft-tissue metastases, the literature largely overlooks the unique clinical complexities of the skeleton [18,19,20,21,22]. Currently, there is no synthesis focused on the distinctive biomechanical, surgical, and prognostic demands of bone metastasis, where decisions hinge on structural stability, fracture risk, survival, and functional outcomes. To bridge this gap, this review addresses three critical questions: (1) How are AI models currently applied across diagnostic imaging, lesion segmentation, fracture risk prediction, prognostic modeling, and surgical decision support in bone metastasis? (2) What data sources, validation strategies, and performance metrics are used, and where do methodological limitations constrain generalizability? (3) Which translational barriers—bias, interpretability, workflow integration, and regulation—most impede clinical uptake, and what practical frameworks could enable implementation? By structuring the review around these questions, we aim to provide clear take-home points and a roadmap for clinically meaningful research.

## 2. Methods

### Approach to the Literature

This article is designed as a narrative review rather than a formal systematic or scoping review. Our aim is to provide a conceptual overview of how AI and ML are being applied across key domains of bone metastasis management, rather than to exhaustively catalog all published studies. To inform this overview, we performed targeted searches of MEDLINE (PubMed) and major publisher databases using combinations of the terms “bone metastasis”, “metastatic bone disease”, “artificial intelligence”, “machine learning”, “deep learning”, “radiomics”, “fracture risk”, and “prognostic model”. We focused on recent peer-reviewed studies that (i) applied AI/ML methods to clinically relevant tasks related to bone metastases and (ii) reported quantitative performance metrics or clearly described clinical use-cases. Additional articles were identified through the reference lists of relevant papers and recent reviews. Given the heterogeneity of study designs, inputs, and outcomes, we summarize the literature narratively and organize it according to major clinical themes rather than conducting meta-analysis.

## 3. Overview of Bone Metastasis

Bone metastases are one of the most common complications associated with advanced malignancies. They affect about one million people around the world every year [1,2,3,4,5,6,7]. Their occurrence persists in increasing alongside enhanced survival rates in solid tumors and the aging population. Prostate and breast cancers are the most prevalent causes of skeletal metastases, affecting up to one-third of patients each. Lung, renal, and thyroid cancers are next in line [3,4,5,6]. About 5% of all cancer patients in the United States have bone involvement, which is about 20 cases per 100,000 people each year [3]. In 2–8% of these cases, pathological fractures happen. These cause pain, immobility, and an important impairment in quality of life [7,8]. The worldwide clinical and socioeconomic burden emphasizes the necessity for accurate risk evaluation and personalized management to avert SREs.

The skeleton is a prevalent site for metastasis due to its broad vascular supply and microenvironment that supports tumor cell viability [1,2,3,4,5,6,7,8,9,10]. The spine, pelvis, and proximal long bones, especially the femur and humerus, are the most common places for metastases to spread [3,11]. These areas are biomechanically important, and a pathological fracture in this region can greatly limit the capability to perform daily tasks. The usual signs and symptoms are worsening bone pain, instability in the joints, and, in more severe cases, neurological problems caused by stresses on the spine and nerves. Diagnosis frequently transpires in the later stages of the disease as initial lesions may be asymptomatic. Improvements in cross-sectional imaging, especially CT, MRI, and PET/CT, have made it significantly simpler to detect lesions and plan treatments [14,15,16].

From a clinical perspective, bone metastases are typically classified as osteolytic, osteoblastic, or mixed, depending on their radiographic characteristics and patterns of bone remodeling [1,2,3,4,5,6]. Osteolytic lesions, prevalent in breast and lung cancers, lead to structural degradation and an increased risk of fractures, while osteoblastic lesions, characteristic of prostate cancer, display disorganized new bone formation that may remain mechanically fragile [3,4,5,6]. Mixed lesions are common in advanced disease and make it harder to plan medical treatment because their structure changes. It is important to understand these patterns in order to predict when mechanical failure will happen, make decisions about surgical procedures, and choose the right systemic therapy, like bisphosphonates or RANKL inhibitors.

Precise evaluation of fracture risk continues to be fundamental to clinical management. The Mirels score, which examines the lesion’s location, size, shape, and pain, is still the most common way to predict pathological fractures [16]. Nevertheless, its dependence on subjective interpretation and categorical variables makes it less accurate and less consistent between observers. Consequently, certain low-risk lesions may fracture unexpectedly, whereas others may experience unwarranted fixation. Current practice increasingly favors a multidisciplinary approach that incorporates radiological, oncological, and orthopedic perspectives. New computational and AI models look promising for making objective, patient-specific risk predictions by looking at imaging features and biomechanical parameters. This could help healthcare providers formulate more effective choices regarding metastatic bone disease.

## 4. Fundamentals of Artificial Intelligence

Artificial intelligence (AI) refers to computer systems that can perform tasks that normally require human cognitive abilities, such as perception, reasoning, and decision-making. Within AI, machine learning (ML) is the principal paradigm through which algorithms learn patterns from data and improve their performance over time without being explicitly programmed for each task [22]. Classical ML approaches are often categorized as supervised learning, in which models are trained on labeled datasets to predict specific outcomes (e.g., fracture occurrence or tumor classification), and unsupervised learning, which identifies latent structures or clusters in unlabeled data. Reinforcement learning constitutes a third paradigm, enabling adaptive optimization through iterative feedback, and has been explored in areas such as surgical robotics and treatment planning. Deep learning (DL), a specialized subset of ML, employs multilayer neural networks to automatically extract hierarchical features from complex inputs, achieving state-of-the-art performance in image and signal interpretation [22].

In musculoskeletal oncology, several AI subfields have particular relevance. Radiomics involves the high-throughput extraction of quantitative imaging features—such as shape, texture, and intensity—from CT, MRI, or PET scans, thereby converting medical images into structured data for diagnosis, prognostication, and treatment response prediction [23]. Natural language processing (NLP) can, in principle, be used to automatically extract clinically relevant information from electronic health records (EHRs), radiology reports, and operative notes, facilitating large-scale data curation and outcome surveillance. However, in the specific context of bone metastases, our review identified only a single NLP-based study, indicating that this remains an emerging rather than established application area. Conceptually, these methods are complementary and may ultimately allow AI systems to integrate imaging-derived and clinical variables into more objective, reproducible, and patient-specific decision-support tools.

A limited number of model architectures have become particularly influential in orthopedic and oncologic imaging. Convolutional neural networks (CNNs) remain the backbone of visual analysis tasks and are widely used for segmentation, classification, and lesion detection through spatial feature learning [23]. Transformers, originally developed for sequence-oriented NLP tasks, have recently been adapted for medical imaging and multimodal data fusion owing to their capacity to model long-range dependencies and contextual relationships [23]. Generative adversarial networks (GANs) are employed to enhance image quality, generate realistic synthetic data for model training, and perform domain adaptation between different imaging modalities. Ensemble models, which aggregate predictions from multiple algorithms, can improve robustness and generalizability—an important property in rare orthopedic tumors and metastatic bone disease, where datasets are often small, heterogeneous, and imbalanced.

AI applications in musculoskeletal oncology ultimately depend on the integration of multiple data types. Imaging data—CT for bone architecture, MRI for soft-tissue delineation, and PET for metabolic activity—form the core of most current models. These are complemented by clinical and laboratory variables such as demographics, comorbidities, primary tumor type, systemic treatment, and biochemical markers, which provide essential context. Structured EHR entries and, where available, NLP-derived variables can support longitudinal outcome tracking and model validation. In practice, however, truly multimodal fusion remains technically challenging: imaging data are high-dimensional and spatial, whereas clinical data are tabular, sparse, and often incomplete, and their temporal alignment is seldom standardized. Based on the analysis of the included literature, we observe that most bone metastasis studies to date either use unimodal models or simple concatenation of feature sets [23,24,25,26,27,28,29,30,31,32,33,34,35,36,37,38,39,40,41,42,43,44,45,46,47,48,49,50,51,52,53,54,55,56,57,58,59,60,61,62,63,64]. The convergence of more advanced multimodal architectures with rigorous preprocessing and harmonization pipelines offers a future pathway toward comprehensive, data-driven decision-making that meaningfully augments clinical judgment in the diagnosis, staging, and management of bone metastases.

## 5. Applications of AI in Bone Metastasis

Artificial intelligence (AI) has emerged as a pivotal component in the multidisciplinary management of bone metastases. Its applications range from the automated detection of lesions in diagnostic imaging to the complex prediction of survival outcomes and fracture risks. To facilitate a structured analysis, we have partitioned the literature into five subsections that mirror the clinical workflow. Section 5.1 focuses on diagnostic and classification models used for lesion identification. Section 5.2 examines segmentation and quantification techniques for assessing tumor burden. Section 5.3 addresses the critical challenge of fracture risk prediction and biomechanical stability. Section 5.4 explores prognostic modeling regarding patient survival and outcomes. Finally, Section 5.5 discusses systems designed for surgical decision support (Table 1, Table 2 and Table 3).

### 5.1. Diagnostic Imaging and Detection

Twenty-five studies investigated AI-based diagnostic imaging for the detection of bone metastasis, utilizing modalities such as bone scintigraphy (BS), Computed Tomography (CT), Magnetic Resonance Imaging (MRI), Positron Emission Tomography (PET), and Single-Photon Emission Computed Tomography (SPECT) (Table 1). Early deep learning implementations predominantly utilized Convolutional Neural Networks (CNNs), such as ResNet and DenseNet, to process 2D image slices or maximum intensity projections. However, recent literature since 2023 indicates a shift toward Transformer-based architectures—including Vision Transformers (ViT), DeiT, and Swin Transformer—which have demonstrated superior capability in extracting global dependencies from heterogeneous datasets [23]. To address the challenge of data scarcity and modal incompleteness, Generative Adversarial Networks (GANs) have been employed to synthesize missing modalities, such as generating synthetic PET images from CT data to enhance lesion conspicuity without additional radiation exposure [25]. While these models demonstrate high internal accuracy, widespread clinical adoption is currently hindered by a lack of external validation. Furthermore, the “black box” nature of deep learning remains a concern; clinical trust requires explainable workflows (e.g., gradient-weighted class activation mapping [Grad-CAM]) to verify that the model is identifying pathological features rather than imaging artifacts.

### 5.2. Lesion Segmentation and Quantitative Assessment

Automated segmentation serves as the fundamental bridge between qualitative imaging and quantitative assessment (Table 2). Unlike simple detection (bounding boxes), segmentation delineates the precise voxel-wise boundaries of a lesion. This step is critical because it isolates the region of interest (ROI) required to extract quantitative radiomic features—such as texture heterogeneity and shape sphericity—which are subsequently used to train machine learning (ML) classifiers.

Data Preparation and Inputs: Reviewers and practitioners must note that raw imaging data is rarely fed directly into these models. Typical preprocessing pipelines for segmentation involve

1. Resampling to an isotropic voxel spacing to standardize physical dimensions.
2. Intensity normalization (e.g., clipping Hounsfield Units in CT) to reduce scanner variability.
3. Noise reduction filters. Once prepared, these volumetric data are processed by architectures such as U-Net, BMSMM-Net [59], or MFP-YOLO [60].

Recent advancements have moved beyond simple geometric segmentation. Advanced frameworks now incorporate multi-attention mechanisms [61,62] to handle the significant variation in lesion size and cortical destruction patterns. By standardizing the delineation of tumor burden, these tools improve the reproducibility of response assessment criteria (e.g., RECIST 1.1) and facilitate precise radiotherapy planning.

### 5.3. Fracture Risk Prediction

AI-based fracture risk stratification represents a crucial, albeit nascent, frontier. Current clinical practice relies heavily on the Mirels score, a scoring system that has been criticized for low specificity, often leading to unnecessary prophylactic stabilizations. An “AI-based fracture risk model” in this context is defined as a system that integrates imaging features with biomechanical simulations. Proof-of-concept studies have successfully coupled Finite Element Analysis (FEA) with deep learning. For instance, spatio-temporal neural networks have been trained to predict the structural deterioration of bone under physiological loads [35]. By utilizing segmentation-derived cortical geometry to calculate local stress concentrations [46], these models aim to provide a personalized, quantitative alternative to the subjective Mirels score. However, this field requires rigorous validation against longitudinal datasets containing confirmed fracture events to transition from theoretical modeling to clinical utility.

### 5.4. Prognostic Modeling and Multimodal Integration

Prognostic modeling has seen rapid growth, utilizing ML to predict survival, disease progression, and treatment response (Table 3). These models frequently employ algorithms such as Random Forest, Support Vector Machines (SVM), and Gradient Boosting (XGBoost/LightGBM). Challenges in Multimodal Integration: A significant challenge in this domain is the integration of “multimodal” data—specifically, combining high-dimensional imaging data (pixels/voxels) with low-dimensional clinical tabular data (e.g., age, primary tumor histology, ALP levels). Unlike imaging features, clinical variables are often sparse or categorical. Recent approaches utilize “late fusion” techniques, where image features extracted via CNNs are concatenated with clinical vectors prior to the final classification layer [37,53]. Notable examples include the PARITY trial analysis, where explainable ML models utilized clinical variables to predict one-year mortality and metastasis risk following endoprosthetic reconstruction [49]. These models prioritize interpretability, allowing clinicians to understand which specific factors (e.g., hemoglobin levels, tumor size) drove the prediction, a requirement for personalized treatment stratification.

### 5.5. Surgical Decision Support and NLP

Beyond imaging and prognosis, AI is increasingly supporting surgical strategy. Applications include predicting quality-of-life improvements post-surgery [47] and optimizing implant selection based on recurrence risk. While traditional ML dominates this space, Natural Language Processing (NLP) is an emerging tool for data curation. Groot et al. [63] demonstrated that NLP could process unstructured radiology reports to distinguish between single and multiple metastases with high sensitivity. Although currently sparse, the integration of NLP to mine electronic health records (EHR) represents a promising avenue to generate the large-scale registries needed to train robust surgical decision algorithms.

## 6. Discussion

The effective management of bone metastasis mandates accurate diagnosis, personalized risk evaluation, and informed surgical decision-making. This review demonstrates that the majority of current research on Artificial Intelligence (AI) and Machine Learning (ML) in this domain utilizes imaging studies for diagnostic tasks such as lesion detection, segmentation, and radiomics-based classification [20,21,23,24,25,28,29,30,31,32,33,34,38,39,40,41,42,43,44,45]. While Convolutional Neural Networks (CNNs) and transformer-based architectures have demonstrated significant accuracy across CT, MRI, PET, and SPECT modalities, Generative Adversarial Networks (GANs) have emerged as powerful tools for synthetic imaging and cross-modality enhancement [23,24,25,29,42,43,44,45].

Beyond diagnostics, a burgeoning domain exists in prognostic modeling and surgical decision support. These applications utilize ensemble methods and explainable machine learning to forecast survival, recurrence, and postoperative quality of life [41,47,49,50,51,52,53,54,55]. However, despite robust internal validation, most studies lack external testing or prospective validation, thereby constraining their generalizability [20,21]. Overall, while AI possesses significant potential to refine the diagnosis of metastatic bone disease and individualized treatment strategies, its translation into real-world clinical practice remains in the nascent stages [20,21,41].

### 6.1. Limitations of the Evidence Base

Several critical limitations within the current literature must be acknowledged. First, the predominance of retrospective study designs severely limits the ability to infer causality or assess real-world clinical impact. Second, most datasets are institution-specific and lack demographic diversity, increasing the risk of algorithmic overfitting and bias. Third, the methodological inconsistency—ranging from variable sample sizes to inconsistent reporting of performance metrics—impedes robust meta-analytic synthesis. Fourth, the literature is heavily skewed toward imaging applications, whereas biomechanical modeling, intraoperative guidance, and long-term functional prediction remain largely unexplored. Finally, there is a notable paucity of health economic evaluations; understanding the cost-effectiveness of these tools is a prerequisite for policy-level integration into oncology care systems.

### 6.2. Diagnostic, Predictive, and Surgical Applications

Diagnostic Imaging and Segmentation: Significant progress has been achieved in diagnostic imaging, where deep learning models frequently outperform traditional radiological interpretation in sensitivity, particularly within bone scintigraphy, CT, and PET datasets [23,24,25,30,31,32,33,38,39,40,42,43,44,45]. It is important to note that raw imaging data—such as CT slices—are rarely fed directly into these classifiers. Instead, data typically undergo rigorous preprocessing, including normalization, noise reduction, and region-of-interest extraction, before being processed by architectures such as CNNs or Support Vector Machines (SVMs). Segmentation models, such as BMSMM-Net and MFP-YOLO, have automated the delineation of lesions, reducing inter-observer variability. This automation is the foundational step for quantitative tumor burden assessment and radiotherapy planning [58,59,60,61,62].

Fracture Risk Prediction: Fracture risk prediction represents a critical yet early-stage frontier. In this context, an “AI-based fracture risk model” is defined as a computational system that integrates imaging features (e.g., cortical thickness, lytic lesion volume) with biomechanical data. Recent proof-of-concept studies have coupled Finite Element Analysis (FEA) with ML frameworks to model osteolytic progression and simulate bone strength degradation under physiological loads [14,15,35]. These “hybrid” models aim to provide a mechanistic, quantitative assessment of stability, offering a superior alternative to purely qualitative guidelines.

Prognostic Modeling: Prognostic modeling—utilizing algorithms such as Random Forest, Gradient Boosting, or LASSO regression—has exhibited predictive efficacy for survival and recurrence, particularly within lung and prostate cancer populations [41,50,51,52,53,54,55]. Notably, the integration of distinct data types remains a challenge. “Clinical data” in these studies refers to tabular variables (e.g., patient demographics, serum biomarkers, primary tumor histology) that are conceptually distinct from imaging features. The challenge lies in correlation; clinical variables may hold prognostic value independent of the radiological appearance of the bone lesion.

### 6.3. Challenges in Multimodal Data Integration

A recurring theme in recent literature is the utility of “multimodal data.” However, integrating fundamentally different data types—specifically high-dimensional, unstructured imaging data (pixels/voxels) and low-dimensional, structured clinical data (tabular)—presents significant technical hurdles. The primary challenge is data heterogeneity; imaging data is dense and continuous, whereas clinical data is often sparse and categorical. Furthermore, clinical datasets frequently suffer from missing values, requiring sophisticated imputation strategies that do not introduce bias into the combined model. To address these issues, current cutting-edge research employs “Late Fusion” or “Intermediate Fusion” strategies. In these frameworks, deep learning models (like CNNs) first extract feature vectors from images, which are then concatenated with clinical variables before passing through the final classification layers, allowing the model to weigh both radiological and clinical factors simultaneously.

### 6.4. Translational Barriers and Interpretability

The translation of AI from code to clinic faces significant barriers regarding bias, interpretability, and workflow integration. Algorithmic bias remains a concern, as training datasets often inadequately represent diverse patient demographics, potentially leading to disparities in clinical performance across different populations. A major impediment to clinical adoption is the lack of explainability; many deep learning models operate as “black boxes,” providing predictions without transparent reasoning. This opacity makes it difficult for clinicians to identify potential failure modes. To address this, the application of Explainable AI (XAI) techniques, such as SHAP (SHapley Additive exPlanations) and Grad-CAM, is essential to visualize the specific features driving a model’s prediction [17,20,21]. Finally, successful deployment requires models to be embedded within Picture Archiving and Communication Systems (PACS) and Electronic Health Records (EHRs), allowing for real-time interpretation without disrupting the clinical workflow.

### 6.5. Clinical Translation in Practice

Bridging the gap between AI research and orthopedic oncology practice demands clinically aligned integration across diagnostic, operative, and follow-up pathways.

#### 6.5.1. Preoperative Assessment and Patient Selection

AI-based systems offer the potential to refine early diagnosis and treatment planning. Automated image-analysis algorithms can rapidly identify cortical breaches or osteolytic zones, enabling the early recognition of mechanically unstable sites [23,24,39,40]. When these imaging features are combined with independent clinical factors, AI can generate individualized risk scores. These quantitative tools serve to complement expert clinical judgment, particularly in the triage of borderline lesions where traditional systems, such as the Mirels score, suffer from low specificity and high inter-observer variability [11,16].

#### 6.5.2. Surgical Planning and Intraoperative Decision-Making

Finite-element-based simulations derived from imaging data allow for the estimation of residual bone strength, facilitating personalized implant selection. Furthermore, AI-supported navigation systems in complex pelvic or spinal resections can improve trajectory accuracy and minimize radiation exposure [44,45,47,65].

#### 6.5.3. Postoperative Surveillance

Longitudinal monitoring is crucial for detecting recurrence. ML models analyzing sequential imaging can identify subtle implant loosening or local recurrence earlier than standard review [38,39,53]. Additionally, Natural Language Processing (NLP) frameworks enable the automated extraction of structured data from unstructured radiology and operative reports, streamlining large-scale outcome evaluation [63].

### 6.6. Future Directions

To advance the field, future research must prioritize the development and validation of a standardized, AI-augmented alternative to the Mirels score. A robust, validated AI scoring system could significantly reduce unnecessary prophylactic surgeries while preventing catastrophic fractures. To achieve this, the field must move toward standardized reporting by adhering to frameworks like TRIPOD-AI and CONSORT-AI, ensuring transparency and reproducibility. Additionally, the adoption of Federated Learning is crucial; utilizing distributed training across multicenter datasets will help overcome privacy barriers and improve model generalizability. Ultimately, the field must transition from retrospective reviews to pragmatic, prospective trials integrated into routine oncology care pathways.

### 6.7. Take-Home Messages

Artificial Intelligence and Machine Learning have demonstrated significant efficacy in diagnostic imaging through CNNs, transformers, and radiomics, facilitating earlier lesion detection and precise segmentation. Beyond diagnostics, emerging fracture risk prediction models and prognostic tools offer a path toward objective, patient-specific alternatives to subjective scoring systems like the Mirels score. However, the majority of current evidence remains retrospective and single-center. Establishing true clinical utility requires multicenter data sharing, standardized reporting (TRIPOD-AI), and prospective validation. Furthermore, clinical implementation mandates rigorous attention to algorithmic bias and the use of Explainable AI (XAI) to ensure transparency. AI is positioned to enhance, not replace, clinical expertise, fostering data-driven care for patients with metastatic bone disease.

## 7. Conclusions

The application of machine learning in the management of bone metastases is a rapidly expanding field, currently dominated by diagnostic imaging capabilities. While surgical decision support and fracture risk prediction remain in their early stages, they hold transformative potential. The evidence suggests a clear imperative to standardize methodologies, validate models via external multicenter cohorts, and integrate these tools into real-world clinical pathways to improve patient outcomes and optimize resource utilization.

## Figures and Tables

**Table 1:** Diagnostic Imaging and Detection Models.

No.	Authors (Year)	AI/ML Methodology	Modality	Objective	Key Findings
[23]	Pak et al. (2025)	CNNs (ResNet, ConvNeXt) vs. Transformers (Swin, ViT)	Bone Scans	Compare architecture performance	Transformers showed superior feature extraction.
[24]	Zhang et al. (2025)	Deep Learning (BLDS System)	CT	Automated detection system	Effective automatic detection pipeline.
[25]	Chai et al. (2025)	GANs (Pix2pix, CycleGAN)	CT → Synthetic PET	Data augmentation	Generated high-fidelity synthetic PET images.
[26]	Oya et al. (2025)	Automated Analysis Software (VSBONE)	Bone Scintigraphy	Diagnostic accuracy evaluation	High diagnostic accuracy for breast/prostate BM.
[27]	Wang Y et al. (2024)	Custom CNN	SPECT	Automated diagnosis	High reliability and accuracy.
[29]	Lin et al. (2024)	GAN with multi-receptive field	SPECT	Synthetic data generation	Effective augmentation for small datasets.
[30]	Elfarra et al. (2019)	Probabilistic Classification	Bone Scintigraphy	Lesion classification	Accuracy 87.58%.
[31]	Koizumi et al. (2017)	Artificial Neural Network (ANN)	Bone Scintigraphy	Detect prostate BM	Sensitivity 82%, Specificity 83%.
[32]	Zhao et al. (2020)	Deep Neural Network (DNN)	Bone Scintigraphy	Skeletal metastasis diagnosis	ROC AUC 0.955–0.988.
[33]	Papandrianos et al. (2020)	CNN	Bone Scintigraphy	Detect prostate BM	Accuracy 91.42%.
[43]	Lin et al. (2021)	CNN	Thoracic SPECT	Automatic detection	Accuracy 0.98, AUC 0.99.
[44]	Noguchi et al. (2022)	Deep Learning	CT	Automated detection	Sensitivity ~89%.

**Table 2:** Lesion Segmentation and Quantitative Assessment.

No.	Authors (Year)	Methodology & Inputs	Modality	Objective	Key Findings
[28]	Ma X et al. (2024)	Dual-path DL (Encoder–decoder + MacBERT)	SPECT	Segmentation refinement	Enhanced segmentation of indistinct lesions.
[34]	Yang et al. (2025)	ML (Extra Trees, CatBoost) + nnUNet segmentation	Multiparametric MRI	Predict BM & Gleason score	Accurate prediction using MRI radiomics.
[38]	Edelmers et al. (2024)	U-Net Architecture	CT	Spinal lesion segmentation	Successful for both osteolytic & sclerotic types.
[39]	Zeng et al. (2024)	DenseNet-264 + Radiomic feature extraction	CT	Predict lung cancer BM	High prediction accuracy.
[40]	Zhao et al. (2024)	DL (MISSU) + SVM-based Radiomics	CT	Tumor differentiation	Effective differentiation of breast cancer BM.
[42]	Murata T et al. (2024)	U-Net (Deep Learning)	Bone Scintigraphy	Image quality enhancement	Improved quality of low-count scans.
[46]	Jakubicek et al. (2019)	CNN + Geometric Analysis	3D CT	Vertebra detection & compression risk	Accuracy 87.1%; identified spinal cord compression.
[58]	Bauckneht et al. (2025)	Radiomics + ML Classifiers	PET/CT	Lesion classification	Distinguishes metastasis from nonspecific uptake.
[59]	Shang et al. (2025)	BMSMM-Net (Mamba architecture)	CT	Precise segmentation	High segmentation accuracy (Dice score).
[60]	Lu et al. (2025)	MFP-YOLO (Transformer decoder)	CT	Multi-scale detection	High accuracy and processing speed.
[61]	Xie et al. (2025)	Encoder–decoder + Multi-attention ViT	SPECT	Automated segmentation	State-of-the-art segmentation results.
[62]	Ma et al. (2025)	CNN + Interactive Attention Module	SPECT	Interactive segmentation	Enhanced user interaction and boundary definition.
[64]	Lin et al. (2022)	Region Proposal Network (RPN)	SPECT	Automatic segmentation	Dice Similarity Coefficient (DSC) 0.692.

**Table 3:** Prognostic Modeling, Clinical Prediction, and Multimodal Integration.

No.	Authors (Year)	Methodology	Data Sources	Objective	Key Findings
[35]	Wang et al. (2025)	Spatio-Temporal NN + FEA	Micro-CT (Preclinical)	Forecast Structural Deterioration	Effective Mechanical Property Prediction.
[36]	Li et al. (2025)	Swin Transformer (Dual-modality)	CT + Pathology images	Predict BM risk in lung cancer	Multimodal fusion improved prediction over single modality.
[37]	Gündoğdu et al. (2025)	XGBoost	MRI + Clinical Data	BM prediction in prostate cancer	Combined data enhances accuracy.
[41]	Rossi et al. (2024)	ML with LASSO selection	Clinical + Lab Data	Predict survival post-radiotherapy	Reliable survival prediction model.
[47]	Kuijten et al. (2025)	ML (RF, SVM, LR, NN)	Clinical Data	Predict post-op Quality of Life	Accurate QoL improvement prediction.
[48]	Yao et al. (2025)	Ensemble ML (XGBoost, RF, AdaBoost)	SEER Clinical Data	Predict BM in thyroid carcinoma	High prediction accuracy.
[49]	Deng et al. (2025)	Explainable ML (SHAP, Boruta, LightGBM)	Clinical Data (PARITY trial)	Predict 1-year mortality	Models were both accurate and interpretable.
[50]	Zhou et al. (2025)	ML (Elastic Net) + DL	SEER Data	Survival nomogram development	Nomogram improved clinical decision-making.
[51]	So et al. (2024)	ML Risk Modeling	Clinical Data	Predict BM risk	High predictive power.
[52]	Jin et al. (2024)	ML (LR, RF, KNN, XGBoost)	Clinical Data	Predict BM in prostate cancer	Effective predictive model.
[53]	Guo et al. (2024)	3D CNN + Clinical Data Fusion	CT + Clinical Data	Predict BM-free survival	Multimodal integration yielded highest accuracy.
[54]	Li et al. (2024)	Random Forest	Clinical Data	Predict castration resistance	Successful classification.
[55]	Thio et al. (2019)	Random Forest, SVM, NN	Clinical Data	Predict 90-day/1-yr survival	Developed robust survival models.
[56]	Liu et al. (2020)	Random Forest, XGBoost	Clinical Data	Predict BM in thyroid/prostate	RF and XGB models showed highest accuracy.
[57]	Zhou et al. (2023)	Logistic Regression, RF, GBM	Clinical Data	Predict BM in lung adenocarcinoma	Comparable AUC across models.
[63]	Groot et al. (2020)	Natural Language Processing (NLP)	Radiology Reports	Differentiate single vs. multiple BM	Sensitivity 0.94, Specificity 0.82.

## Data Availability

No new data were created or analyzed in this study.

## Abbreviations

The following abbreviations are used in this manuscript:

AI	artificial intelligence
CNN	convolutional neural network
CONSORT-AI	Consolidated Standards of Reporting Trials—Artificial Intelligence extension
CT	computed tomography
EHR	electronic health record
FEA	finite element analysis
GAN	generative adversarial network
LASSO	least absolute shrinkage and selection operator
ML	machine learning
MRI	magnetic resonance imaging
NLP	natural language processing
NSCLC	non-small cell lung cancer
PACS	picture archiving and communication system
PARITY	Prophylactic Antibiotic Regimens in Tumor Surgery trial
PET	positron emission tomography
PRISMA-ScR	Preferred Reporting Items for Systematic Reviews and Meta-Analyses Extension for Scoping Reviews
SEER	Surveillance, Epidemiology, and End Results
SHAP	SHapley Additive exPlanations
SPECT	single-photon emission computed tomography
SRE	skeletal-related event
TRIPOD-AI	Transparent Reporting of a multivariable prediction model for Individual Prognosis Or Diagnosis—Artificial Intelligence extension
ViT	Vision Transformer
